# The evaluation of a Taiwanese training program in smoking cessation and the trainees' adherence to a practice guideline

**DOI:** 10.1186/1471-2458-10-77

**Published:** 2010-02-18

**Authors:** Fei-Ran Guo, Ling-Yu Hung, Chih-Jen Chang, Kai-Kuen Leung, Ching-Yu Chen

**Affiliations:** 1 Department of Family Medicine, National Taiwan University Hospital and College of Medicine, Taipei, Taiwan; 2 Department of Family Medicine, Cardinal Tien Hospital Yung Ho Branch, Taipei, Taiwan; 3 Department of Family Medicine, National Cheng Kung University Hospital and College of Medicine, Tainan, Taiwan; 4 Division of Geriatric Research, Institute of Population Health Sciences, Department of Family Medicine, National Taiwan University Hospital and College of Medicine, Taipei, Taiwan

## Abstract

**Background:**

The Taiwanese government began reimbursement for smoking cessation in 2002. Certification from a training program was required for physicians who wanted reimbursement. The program certified 6,009 physicians till 2007. The objective of this study is to evaluate the short- and long term efficacy of the training program.

**Methods:**

For short term evaluation, all trainees in 2007 were recruited. For long term evaluation, computer randomly selected 2,000 trainees who received training from 2002 to 2006 were recruited. Course satisfaction, knowledge, confidence in providing smoking cessation services and the adherence to a practice guideline were evaluated by questionnaires.

**Results:**

Trainees reported high satisfaction with the training program. There was significant difference between pre- and post-test scores in knowledge. Confidence in providing services was lower in the long term evaluation compared to short term evaluation. For adherence to a practice guideline, 86% asked the status of smoking, 88% advised the smokers to quit, 76% assessed the smoker's willingness to quit, 59% assisted the smokers to quit, and 60% arranged follow-up visits for smokers. The incentive of reimbursement was the most significant factor affecting confidence and adherence.

**Conclusions:**

The training program was satisfactory and effective. Adherence to a practice guideline in our study was better than studies without physician training in other countries.

## Background

Smoking cessation is the most effective intervention to improve health outcomes. According to a cohort study recruiting 66,161 Taiwanese [1], the relative risk of all cause mortality was 1.55 for male smokers and 1.89 for female smokers. The relative risk of cancer mortality was 1.67 for male smokers and 2.42 for female smokers. Smoking also increased mortality in diabetes, cardiovascular, respiratory, digestive system, and renal diseases, as well as accidents and suicide. In 2001, 18,803 deaths, or one out of four deaths, in middle aged Taiwanese men were attributable to smoking [2].

There are 4.8 million smokers in Taiwan and 47% of them have ever considered quitting [3]. The smoking rate for Taiwanese males was greater than 60% two decades ago, while the female smoking rate was around 3-4%. After tremendous efforts by the government and society to fight tobacco use, the male smoking rate decreased to 47% [4]. The government began collecting a tobacco tax in 2002 providing more revenues for use in tobacco control.

An outpatient-based smoking cessation service was proposed by the Bureau of Health Promotion (BHP). Physicians providing services were reimbursed for counseling and patients received partial payment of medications. However, most physicians did not have the knowledge in smoking cessation and were not familiar with consultation skills. A joint-committee of family medicine physicians, internal medicine physicians and psychiatrists proposed a practice guideline. The guideline was based on the United States Public Health Service (US PHS) guideline published in 2000 [5], including the 5As (ask, advise, assess, assist, arrange), brief counseling by physicians, pharmacotherapy, and relapse prevention [6].

To ensure quality of care, BHP sponsored the Taiwan Association of Family Medicine (TAFM) to offer a nationwide training program for physicians. The 6-hour training was mandatory for physicians who wanted to receive reimbursement. Till December 2007, there were a total of 6,009 physicians certified. There are about 35,000 physicians in Taiwan, which means one in six physicians received smoking cessation training. In a report in 2007 [7], there were 3,887 physicians contracting with BHP and provide smoking cessation services. Most were primary care physicians. There were 250,000 patients that received the services. The six-month quit rate was 22%.

A nationwide survey was conducted in 2007. The purpose of this survey was to assess the quality of the training and the trainees' adherence to the practice guideline. In this paper, we present the results of this survey. To our knowledge, this is the first nationwide smoking cessation training program with very high rate of physician participation.

## Methods

### The contents of the training program

An expert committee composed of fourteen delegates from TAFM, Taiwan Society of Internal Medicine, Taiwanese Society of Psychiatry, and BHP proposed training contents and monitored the quality of the program. The training was administered in one day and included five topics: the trans-theoretical model, the clinical skills for smoking cessation, the pharmacotherapy of smoking cessation, the strategy of tobacco control and clinical case discussion. Two additional topics were added in 2007: nicotine addiction and withdrawal, and the risks of smoking and the benefits of quitting. The training was 6 hours plus one hour of explaining the process of contracting with BHP and the administration of reimbursement.

### Subjects

For short term evaluation, all trainees in 2007 were recruited. For long term evaluation, 2000 trainees, who participated during 2002 to 2006, were randomly selected from 5,478 trainees by a computer program.

### Short term evaluation of the training program

Two self-reported, structured questionnaires were used for short term evaluation. The first questionnaire contained three parts: trainee satisfaction, trainee confidence in providing services after training, and the topic the trainees considered most useful. The five-point Likert scale was used to encode satisfaction and confidence levels. The second questionnaire contained 10 multiple-choice questions evaluating knowledge of smoking cessation. The questionnaire was administered before and after the course to measure trainee progress. Both questionnaires were designed by the expert committee. Communication between committee members through e-mails achieved the requirement of face validity of the questionnaires.

### Long term evaluation of the program

A self-reported, structured questionnaire evaluated trainee confidence in providing smoking cessation services and adherence to the 5As guideline. The five-point Likert scale was used to encode confidence levels and the level of adherence. We added the scores of five items related to confidence and that became a 5-to-25 point scale we called "confidence scale." We also added the scores of five items related to adherence to the 5As guideline that became another 5-to-25 point scale we called "adherence scale." These two scales were used to explore the factors affecting confidence and adherence. Questionnaires were mailed to the selected trainees in August 2007. The trainees were asked to return the questionnaire in two weeks. An additional questionnaire was mailed to non-respondents and an additional week used to collect responses. The questionnaire was also designed by the expert committee. Communication between committee members through e-mails achieved the requirement of face validity of the questionnaire.

### Statistics

Descriptive statistics such as frequency, mean and standard deviation were used in variables like satisfaction, confidence and adherence. The Chi-square test was used for examining the difference in demographic data between respondents and non-respondents. Paired t-test was used for detecting the difference between the pre- and post-test. One way ANOVA and t-test were used for the correlation between demographic data and the confidence and adherence scales. Stepwise multiple regression analysis was used for the prediction value of the confidence and adherence scales. Two-tailed test with probability less than 0.05 was used for the rejection of the null hypothesis.

### Ethical Approval

This study was reviewed and approved by the institutional review board of National Taiwan University Hospital.

## Results

### Short term evaluation

There were 531 trainees certified by the training program in 2007. Effective questionnaires were collected from 391 for a response rate of 74%. Among these trainees, 212 (40%) were family physicians, 117 (22%) were internal medicine physicians, 27 (5%) were surgeons, 52 (10%) were pediatricians, 16 (3%) were obstetrics/gynecologists, and 107 (20%) were other specialists. With regard to workplace setting, 127 (24%) worked in medical centers, 144 (27%) in regional teaching hospitals, 61 (12%) in local hospitals, and 199 (38%) were working in private offices or clinics.

Approximately eight out of ten trainees were satisfied or very satisfied with the program. All topics of the program received average scores of more than four out of five points in the evaluating items. The topic trainees considered most useful was the pharmacotherapy of smoking (39%). The percentage of trainees rating other topics as useful were as follows: clinical skills related to smoking cessation (30%), the trans-theoretical model (11%), the risks of smoking and the benefits of quitting (7%), the case discussion (5%), the strategy of tobacco control (5%) and nicotine addiction and withdrawal (4%).

Most trainees were confident or very confident in all evaluating items assessing confidence regarding smoking cessation work directly after the training program. The average score was just over four out of five on all items (Table 1).

**Table 1:** Confidence in providing smoking cessation services after the training, the short term evaluation

	Confident	very confident	Total	average score
Encourage the patients to quit smoking	307 (80)	54 (14)	386 (100)	4.07
Behavioral therapy of smoking cessation	299 (78)	50 (13)	386 (100)	4.03
Pharmacotherapy of smoking cessation	300 (78)	50 (13)	387 (100)	4.03
The confidence in providing services	267 (70)	94 (24)	387 (100)	4.17
Helping patients to quit	237 (61)	105 (27)	387 (100)	4.14

For trainee knowledge, there were 536 effective questionnaires collected from 542 trainees for a response rate of 99%. The mean score of pre-test was 7.14, while the mean score of post-test was 9.22. The mean difference between pre- and post-test scores was 2.08 (SD = 1.79). The paired t-test revealed significant difference (P < 0.001). (Table 2).

**Table 2:** The comparison of pre-post test scores in short term evaluation

	Mean score	SD	t	P
Pre-test	7.14	1.81		
Post-test	9.22	1.12		
Difference	-2.08	1.79	-27.01	0.000*

* P < 0.001

### Long term evaluation

Among 2,000 selected trainees, 770 returned questionnaires during the study period. There were 11 ineffective questionnaires for an effective response rate of 38%.

There were more male (91%) than female participants. The major age group was 40 to 59 years (62%). More respondents trained in 2005 (37%) as compared to 2004 (9%). The majority of trainees were family physicians (52%). No demographic differences were found between responders and non-responders in the long term evaluation with the exception that family physicians were somewhat more likely to be responders as compared with other specialties included in the study.

Table 3 shows the confidence of the respondents in providing smoking cessation services. "Pharmacotherapy of smoking" and "the clinical skills of smoking cessation" were the items respondents were most confident of followed by "the trans-theoretical model" and "the strategy of tobacco control." An interesting finding was the reduced confidence over time. The mean confidence score was above four on the five grade scale in the initial post training assessment for all variables (Table 1). However, in the long-term evaluation no mean confidence score reached four (Table 3).

**Table 3:** Confidence in providing services in long tern evaluation

Confidence in providing services	confident	very confident	Total	average score
Pharmacotherapy of smoking	380 (51)	32 (4)	748 (100)	3.40
Trans-theoretical model	304 (41)	22 (3)	743 (100)	3.29
Clinical skills of smoking cessation	366 (50)	22 (3)	746 (100)	3.38
Strategy of tobacco control	274 (37)	12 (2)	744 (100)	3.20
Administration of services	198 (27)	14 (2)	742 (100)	3.05

The percentages of respondents practicing the 5As guideline for at least half of their patients were as follows: 86% asked about smoking status, 88% advised smokers to quit, 76% assessed the willingness to quit, 59% assisted the smokers to quit, and 60% arranged for follow-up visits (Table 4). The mean scores ranged from 3.90 for advising the smokers to quit to 3.02 for assisting smokers to quit. There were 330 trainees (43%) who performed all 5As for at least half of their patients.

**Table 4:** The adherence to the 5As guideline in long tern evaluation

Adherence to the 5As guideline	none of the patients	very few patients	half of the patients	most patients	almost all patients	Total	average score
Ask the status of smoking	17 (2)	85 (11)	117 (16)	268 (36)	259 (34)	746 (100)	3.89
Advise the smokers to quit	10 (1)	77 (10)	103 (14)	348 (47)	209 (28)	747 (100)	3.90
Assess the willingness of smokers to quit	28 (4)	147 (20)	204 (28)	253 (34)	111 (15)	743 (100)	3.37
Assist the smokers to quit	49 (7)	254 (34)	153 (21)	202 (27)	82 (11)	740 (100)	3.02
Arrange the follow-up visits of smokers	80 (11)	219 (30)	122 (17)	238 (32)	81 (11)	740 (100)	3.03

Demographic data were analyzed to explore possible factors correlating with the confidence and adherence scales (Table 5). The confidence scale had "specialty of physicians" and "contracted with the BHP" as significant factors, while the adherence scale had age and "contracted with the BHP" as significant factors. We used multiple regression models to examine these factors further. The results revealed "contracted with the BHP" and "internal medicine physicians" were significant predictors of the confidence scale, while "contracted with the BHP" was the only significant predictor of the adherence scale. Among these predictors, "internal medicine physicians" had a negative effect. We also studied the correlation between the confidence and adherence scales. The Pearson correlation test revealed significant results (P < 0.001). The internal consistency of these two scales was evaluated. The Cronbach's alpha of confidence scale was 0.88, while the Cronbach's alpha of adherence scale was 0.85.

**Table 5:** The predictors of confidence scale and adherence scale

	Confidence scale Mean ± SD	*P *value	Adherence scale Mean ± SD	*P *value
Age		0.330		0.014*
27-39	16.06 ± 3.89		16.56 ± 3.80	
40-59	16.46 ± 3.26		17.19 ± 4.32	
60-87	16.12 ± 3.04		18.14 ± 4.87	
Specialty		0.046*		0.429
Family medicine	16.36 ± 3.24		16.96 ± 4.35	
Internal medicine	15.74 ± 3.45		17.13 ± 4.09	
Psychiatric	16.89 ± 3.08		16.72 ± 3.43	
Others	16.71 ± 3.07		17.62 ± 4.40	
Contracted with the BHP		< 0.001*		< 0.001*
Yes	16.83 ± 3.12		18.18 ± 3.92	
No	15.07 ± 3.17		14.84 ± 4.39	

Abbreviations: BHP, Bureau of Health Promotion

* P < 0.05

## Discussion

After England, Taiwanese government is the second in the world to reimburse smoking cessation. Smoking cessation services in England do not require specific training in order to receive reimbursement. The advent of smoking cessation treatment services resulted in a rapid proliferation of training courses in smoking cessation over the first few years of implementation. An audit of smoking cessation training suggested that training quality was variable [8]. Smoking cessation training in Taiwan is centralized and sponsored by the government. It is mandatory for all physicians who want to receive reimbursement. The results of this research represent our efforts at promoting quality in the training program.

There was not a standard how a smoking cessation training program should be organized or how many hours physicians should be trained. We complied with the suggestion of US PHS guideline that the physicians provide pharmacotherapy and brief intervention less than 10 minutes in each encounter. Therefore Pharmacotherapy of smoking cessation was an essential topic. The trans-theoretical model and the clinical skills for smoking cessation were the theoretical and practical components of counseling training. We included an hour of clinical case discussion to simulate real world cases and enhanced the interaction between lecturers and audience. The strategy of tobacco control illustrated the global tobacco issues and the policy aspects of tobacco control. Two additional topics were added in 2007: nicotine addiction and withdrawal, and the risks of smoking and the benefits of quitting. We believed the two new topics could enhance the physician's ability in encouraging smokers to quit. People may concern a six-hour training was not enough due to the complexity of behavior-changing counseling. The purpose of our counseling training was to provide the brief intervention during physician's daily practice, not the cognitive-behavior therapy which is usually provided by a psychotherapist. It costs too much to train a physician to provide cognitive-behavior therapy and such training was not suggested by smoking cessation guidelines.

It is not surprising that our program generated high levels of satisfaction and had significant pre- and post-test differences in trainee knowledge. Most of the Taiwanese physicians had little if any previous knowledge on smoking cessation information and skills. There were no courses teaching smoking cessation in medical schools and very few continuing medical education courses focused on smoking cessation. Initially, the National Health Insurance would not cover smoking cessation costs so very few physicians were willing to provide services. The situation changed in 2002 after the barrier of payment was removed. As a result, one in six physicians in Taiwan attended the training program and smoking cessation became a popular service. Critics may argue that one in six is not a large portion of physicians in comparison with other popular training such as hypertension or diabetes. However, the rapid expansion of smoking cessation services already exceeded the expectation of Taiwanese government. The budget was not enough to cover reimbursement in 2008 and the government has raised cigarette tax since June, 2009.

Long term evaluation results revealed that trainee confidence in providing services declined after a period of time. More trainees answered "neutral" or "unconfident" when asked about providing services compared to the short term evaluation. Because there was a significant difference in the confidence between the trainees contracting or not contracting with BHP, we inferred the decline in confidence as possible being due to trainees not providing services. Knowledge and skills of trainees not providing services would decay. A study revealed a similar observation. Physicians actively assisting patients to quit had higher levels of performance and self-efficacy [9]. It is also possible that the trainees under estimated the complexity of smoking cessation service at the time of the short term evaluation. This may have contributed to the decline in confidence at follow-up. This may indicate an initial underestimation of the relative complexity of behavior change support. Similar results have been reported from other studies assessing tobacco prevention work in general practice [10].

The percentages of trainees practicing 5As guideline were higher than those in studies from the US [11-14], China [15], and Hong Kong [16]. However, the high adherence to the guideline was probably due to the payment policy. To remove the influence of payment, we further analyzed the data by extracting the trainees not contracting with BHP from the total trainees. The trainees not contracting with BHP practiced 5As as follows: 75% for ask, 84% for advise, 66% for assess, 38% for assist, and 35% for arrange. These data represented the adherence to 5As guideline without the incentive of payment. The results were still higher than studies in the US (67% for ask, 74% for advise, 35% for assist, 8% for arrange) [13], China (48% for ask, 64% for advise) [15] and Hong Kong (77% for ask, 74% for advise, 20% for arrange) [16]. The adherence levels in our study might be overestimated because respondents may have tried to choose the "right" answer [17,18]. Collecting chart reports would better estimate adherence. The National Ambulatory Medical Care Survey (NAMCS) in the US was an example [12]. In this study, 32% of patient charts did not include information about tobacco use, 81% of smokers did not receive assistance, and less than 2% received a prescription for pharmacotherapy. However, physicians might forget to record this information in charts and the results underestimated actual practice [12]. There is not an ideal method to estimate guideline adherence except through direct observation of encounters, which is difficult to carry out [14].

Physicians with greater self-confidence, female, in private offices, and younger, showed greater compliance with smoking cessation guidelines [13,14,19-21]. However, another study revealed age, gender, practice site (e.g., HMO, solo practice), and pediatric subspecialty were not related to guideline adherence [22]. One study of primary care physicians, who were older, with academic positions, with trained staff for counseling, and with higher confidence, showed they were more likely to advise patients to quit [11]. In our study, physicians contracting with the BHP were more likely to have higher confidence in their ability to provide smoking cessation services and better adherence to the practice guideline. The significant correlation between confidence and adherence could be explained by the confounding effect of payment. Nevertheless, there was a possible causal relationship in that physicians with higher confidence were more adherent to the practice guideline [11,20,22,23]. Internal medicine physicians were less confident than family physicians or psychiatrists in this study. The reason was probably less training in counseling skills. Block *et al. *reported that specialists had fewer counseling skills in smoking cessation than primary care physicians [24]. Adherence to the guideline correlated to physicians' age in this study. The older the physician, the better the adherence to the guideline; however, age was excluded in the multiple regression models. We did not find differences in gender, practice settings (primary care, hospitals, medical centers), stakeholders (private, public), or training years (2002 to 2006) as having association with the adherence to the practice guideline.

The response rate of our long tern evaluation questionnaire (38%) was lower than other studies [11,12,19-23]. It has been well recognized that physicians in Taiwan are reluctant to answer questionnaires. For example, a study investigating physicians' attitudes toward DNR of terminally ill cancer patients in Taiwan had a response rate of 18% [25]. Another study investigating obstetricians' willingness to practice collaboratively with midwives had a response rate of 16% [26]. These were national studies and published in an international journal. To our knowledge, the response rate of our study was high compared to other Taiwanese studies [27-30].

The remaining question was whether the respondents represented a valid sample of the study population. There was no difference in gender, age, and training year between respondents and non-respondents. Another estimation of representativeness was the percentage of the trainees contracting with BHP. There were 70% trainees contracted with BHP in the study population while 70% in our respondents, too. The above analysis revealed that there were not obvious biases in our sample. However, we could not exclude the possibility that physicians who were better in providing smoking cessation services were more likely to respond to the questionnaire.

Evidence suggested the implementation of a smoking cessation practice guideline could decrease smoking rates. Katz *et al*. conducted a non-randomized, before-after trial at family practice settings. After a two-month period of guideline-derived intervention, the test sites had higher two-month and six-month quit rates compared to rates before the intervention while the controlled sites had no difference [31]. Katz and his colleagues conducted another randomized controlled trial with similar design after two years and confirmed the effectiveness of implementing practice guideline [32]. Ward *et al*. analyzed the data from 138 Veterans Administration medical centers suggesting the implementation of the Agency for Health Care Policy and Research (AHCPR) smoking cessation guideline increased smoking cessation counseling and decreased smoking rates [33]. These studies suggested that the adherence to a practice guideline could be a good quality indicator of smoking cessation training programs.

There were several limitations to this study. There was not a control group or baseline data of guideline adherence to verify the effectiveness of training. We compared our data with other studies that were not conducted in Taiwan. The Chinese culture possible made people tend to choose the "right" answer and over-estimated the adherence. It was not a problem when comparing our data with the studies in China and Hong Kong, but could be a problem when comparing our data with studies in other non-Chinese countries. Although the representativeness of respondents in the long term evaluation was acceptable, there were greater numbers of family physicians and fewer psychiatrists. The difference was significant. The response rate to the long term evaluation questionnaire was relatively low, although it was higher when compared to other Taiwanese studies. The low response rate due to Taiwanese culture was understandable, however, it could compromise the quality of the study. The outcome of this study was adherence to the 5As guideline, which served as a surrogate outcome. The evidence of an increased quit rate after the training was not available.

It would be interesting to explore the reasons why 30% of the trainees were not contracting with BHP and not getting reimbursement. Less than 1% of trainees answered "unconfident" or "very unconfident" in providing smoking cessation services in the short term evaluation. The discrepancy between the confidence in providing services, and the practice of providing the services requires further study.

We suggest smoking cessation training should be one part of undergraduate course in medical school. It costs a lot of resources to implement post-graduate training in large scale. There are some medical schools in Taiwan which teach smoking cessation knowledge and technique in undergraduate curriculum. We anticipate more medical schools implementing this policy in the future.

## Conclusions

In this cross-sectional study, we evaluated a nationwide smoking cessation training program for physicians. The short term evaluation revealed high satisfaction among trainees, and significant pre-post test difference in knowledge. The long term evaluation showed that the trainees were more adherent to the 5As guideline than physicians without training in other countries. The decline of confidence in providing smoking cessation services during the long term evaluation is possible due to the trainees increased insight into the complexity of behavior change support after the training course.

## Competing interests

The authors declare that they have no competing interests.

## Authors' contributions

FR designed the study protocol, analyzed the data, and finalized the manuscript. LY helped to analyze the data, and drafted part of the manuscript in Chinese. CJ served as the principle investigator of the training program and monitored the progress of the study. KK served as the consultant of the statistics and helped to modify the manuscript. CY served as the consultant of the training program and the study design. All authors read and approved the final manuscript.

## Pre-publication history

The pre-publication history for this paper can be accessed here:

http://www.biomedcentral.com/1471-2458/10/77/prepub
